# microRNA与肺癌

**DOI:** 10.3779/j.issn.1009-3419.2010.04.21

**Published:** 2010-04-20

**Affiliations:** 650031 昆明，昆明医学院第一附属医院肿瘤实验中心 Cancer Research Center, the First Affiliated Hospital of Kunming Medical College, Kunming 650031, China

目前，我国肺癌的发病率及死亡率迅速增高，已跃居城市人口恶性肿瘤死亡原因的第一位。microRNA（miRNA）是一类新近发现的长约18-25个核苷酸的非编码小分子RNA，是哺乳动物基因组中最为丰富的调节基因之一，可能调控着至少1/3的人类编码蛋白基因^[1-3]^。近年研究证明microRNA的表达失控与肺癌的发生发展密切相关。本文就microRNA在肺癌研究中的进展做一综述。

## 1 microRNA概述

### 1.1 microRNA的形成过程及作用机制

microRNA被认为是从DNA转录、不被翻译但能调控其它基因表达的分子。microRNA的初始转录产物-初微microRNA（pri-miRNA）在细胞核内被核酸酶Drosha加工成miRNA前体（pre-miRNA）^[4]^，然后经Ran-GTP及其受体Exportin-5转运出细胞核^[5, 6]^。60 nt-90 nt的pre-miRNA形成茎环的结构，细胞之中的RNaseIII-Dicer酶将成熟的miRNA从发夹状的pre-miRNA的茎区域中剪切出来，最后成熟的miRNA装配进RNP形成RNA诱导的沉默复合体（RNA induced silencing complex, RISC），执行RNAi相关的基因沉默^[7]^。

microRNA可以指导效应复合物RISC通过两种不同的机制即mRNA剪切和翻译抑制下调靶基因的表达。根据目前公认的观点，microRNA与其靶基因的互补程度决定了它以何种机制沉默靶基因。一旦microRNA组装进入效应复合物RISC，当其与靶mRNA部分互补特别是与3′ UTR的碱基配对后可阻抑翻译；如果其与靶mRNA完全互补，mRNA可被类似siRNA的机制降解。

### 1.2 microRNA的命名原则

研究人员^[8, 9]^对发现的microRNA制定了如下命名规则：①microRNA简写成miR，再根据其被克隆的先后顺序加上阿拉伯数字，如miR-21；②高度同源的microRNA在数字后面加上英文小写字母（a、b、c等），如miR-199a和miR-199b；③由不同染色体上的DNA序列转录加工而成的具有相同成熟体序列的microRNA，则在后面加上阿拉伯数字以区分，如miR-199a-1和miR-199a-2；④如果一个前体的两个臂分别加工产生microRNA，则根据克隆实验在表达水平较低的miRNA后面加“^*^”，如miR-199a和miR-199a^*^，或进行如下命名，如：miR-142-5p（也可命名为miR-142-s，表示从5′端的臂上加工而来）；⑤将物种缩写置于microRNA之前，如has-miR-199；⑥确定命名规则之前发现的microRNA，如let-7等保留原来的名字。

### 1.3 microRNA和siRNA

小RNA分子是非编码的RNA分子，控制着真核细胞的许多功能，影响基因的表达、细胞周期和个体发育等多种行为。小RNA主要包括小干扰RNA（small interfering RNA, siRNA）和微RNA（microRNA）。

siRNA是一种短片段双链RNA分子，能够以同源互补序列的mRNA为靶目标降解特定的mRNA，这个过程也就是RNA干扰（RNA interference, RNAi）。microRNA与siRNA并不相同，但又密切相关；他们之间很容易混淆，有很多共同点也有许多不同点。

主要区别点包括：①来源不同：siRNA通常是外源的，如病毒感染和人工插入的dsRNA被剪切后产生外源基因进入细胞，而microRNA是内源性的，是一种非编码的RNA，由miRNA基因表达出最初的pri-miRNA分子加工后形成；②作用机制不同：siRNA渗入RISC中，引导复合物与miRNA完全互补，通过其自身的解旋酶活性解开siRNA，通过反义siRNA链识别目的mRNA，通过内切酶活性切割目的片段，再经细胞外切酶进一步降解目的片段。siRNA也可以抑制具有短片段互补mRNA的翻译。microRNA通过与miRNP核蛋白体复合物结合，识别靶基因mRNA，并与之部分互补，从而抑制翻译；③生物学意义不同：siRNA不参与生物发育，是RNAi的产物，原始作用是抑制转座子活性与抵御病毒感染。microRNA主要在发育过程中发挥作用，调节内源性基因的表达，具有高度保守性、时序性和组织特异性。

### 1.4 microRNA表达谱高通量分析方法

2002年首次报道了microRNA分子与癌症的关系，研究^[10]^发现癌细胞存在特异的microRNA缺失或表达下调。接着一系列的报道指出在各种癌症中许多microRNA分子的表达都有明显降低，但也有一部分microRNA分子表达是上调的。鉴于这些研究结果，科学家们试图建立起不同癌症的microRNA表达谱库，以期应用于临床诊断。

迄今为止，寻找microRNA分子表达模式的高效率、高通量方法对于研究microRNA分子功能与癌症的关系是非常重要的。目前常用的方法有microRNA克隆、微阵列分析法（microarray analysis）、微珠表达分析和其它方法。由于microRNA分子片段小，在进行高通量表达分析时需要特殊考虑，另外microRNA家族成员序列高度相似性的存在也使问题变得更复杂，因此微阵列的运用已成为最主要的方法。

微阵列分析法实际上是一种微型生物传感器，在不同的基质表面有序地点阵排列了一系列生物分子，固定在每一点阵上的分子都是已知的。在相同条件下，点阵上的分子与其配体分子反应，反应结果用核素、荧光、化学发光或酶标法显示，再通过计算机软件分析，综合成可读总信息，实现对化合物、蛋白质、核酸、细胞或其它生物组分准确、快速、高通量地筛选或检测。microRNA的微阵列分析法目前已广泛用于特异组织或细胞内microRNA表达谱的研究。但对microRNA表达特征的研究是间接的，在分析过程中仍存在特异性和敏感度不高等缺点，因此结果有一定的假阳性，需要辅助其它更为准确的表达研究方法加以验证，如PAGE/Northern blot等^[11]^。

## 2 microRNA和肺癌

Volinia等对123例肺癌组织与123例正常肺组织进行了microRNA的表达差异比较研究，发现有38种microRNA存在表达差异，如let-7家族、miR-16-2、miR-21、miR-191、miR-17-5p以及miR-155等。Yanaihara等利用microRNA基因芯片分析法，对104例非小细胞肺癌组织与正常肺组织研究发现，有43种microRNA有明显的表达差异，15种microRNA发生上调及28种microRNA发生下调。这些发现^[12]^均为microRNA在肺癌中的研究奠定了基础。

### 2.1 let-7与肺癌

let-7最早在线虫中发现，它调控细胞的分化和增殖的时序，成熟的let-7序列在线虫和人类中是保守的。目前发现人类let-7家族有12种，包括let-7a-1、let-7a-2、let-7a-3、let-7b、let-7c、let-7d、let-7e、let-7f-1、let-7f-2、let-7g、let-7i和miR-98。人类let-7家族成员大多定位于9、11、22号染色体上。

Takamizswa^[13]^在2004年通过Northern blot分析了20个人类肺癌细胞株和2个永久人类正常肺上皮细胞株，发现在正常肺上皮细胞株中易检测到成熟let-7，与正常肺组织表达水平相似，60%肺癌细胞株let-7表达水平明显下降。同样在143例原发性肺癌组织标本中也发现let-7表达下调，其中表达水平下降 > 80%的病例占44%，说明let-7在肺癌体内和体外表达均下调。在肺癌细胞系中，let-7家族的过表达能够抑制细胞的增殖、分化和侵袭转移。

let-7的靶基因包括*Ras*家族、高迁移组A蛋白（*HMGA2*）、*c-Myc*、*CDC25A*、*CDK6*和*Cyclin D2*。其中最典型的是促进细胞增殖的*Ras*家族和参与细胞增殖分化的*HMGA2*。*Ras*原癌基因是*Ras*基因家族成员之一，属于膜相关的鸟苷三磷酸酶信号蛋白，可调节细胞生长和分化。HMGA2是与染色体结合的非组蛋白，通过抑制核苷酸切除修复途径引起基因不稳定，促进肿瘤的发展。2005年Johnson等^[14]^证实肺癌中let-7的降低使其调控的靶点*Ras*基因过表达。let-7在肺癌的低表达和Ras的高表达提示microRNA可以作为抑癌基因。研究发现HMGA2在一些恶性肿瘤中如乳腺癌、非小细胞肺癌、胰腺癌表达增高，对恶性肿瘤的生长、分化和转移起重要作用。Mayr等研究发现let-7主要依赖于HMGA2的3′UTR，由于染色体移位毁坏了HMGA2的3′UTR结构，无法与let-7结合而失去对let-7的抑制作用，引起以不依赖支持物生长为特征的致瘤性转化。

### 2.2 microRNA-29家族与肺癌

目前认为肿瘤的发生和发展同时受遗传学和表观遗传学的调控。表观遗传学只影响表型不改变基因型，包括DNA甲基化改变、组蛋白修饰和非编码RNA诱导的改变。基因组甲基化的改变受DNA甲基转移酶（DNA methyltransferase, DNMT）的调控，DNMT催化S腺苷甲硫氨酸的甲基基团转移至胞嘧啶环的5′端。DNMT3A和DNMT3B在肺癌中也经常出现上调，且DNMT3A和DNMT3B出现上调的肺癌预后差。一些miRNA在肺癌中表达下调，其中miR-29家族（miR-29a、miR29b和miR29c）与DNMT3A和DNMT3B的3′UTR互补。Fabbri等^[15]^将DNMT1、DNMT3A和DNMT3B的互补位点克隆至荧光素酶基因的3′UTR中，然后与miR-29a、miR29b和miR29c共转染入肺癌A549细胞。结果显示总DNA甲基化水平与对照组相比有明显下降，转染48 h后下降30%，72 h后下降40%，H1299细胞也有类似情况。因此在肺癌组织中miR-29的表达与DNMT3A和DNMT3B呈负相关，miR-29直接靶定DNMT3A和DNMT3B。Fabbri等还发现在肺癌细胞株中表达miR-29可以修复异常的DNA甲基化，诱导甲基化相关沉默肿瘤抑制基因的重新表达，抑制肿瘤生长。Rajewsky^[16]^也认为DNMT1、DNMT3A和DNMT3B可能都是miRNA潜在的靶点。

### 2.3 miR-34家族与肺癌

*p53*是经典的抑癌基因，在多种肿瘤相关应激信号的刺激下被激活，引起细胞周期阻滞、促进DNA修复、诱导细胞衰老死亡等生物学效应。近期研究^[17, 18]^发现miR-34家族作为*p53*靶基因参与传统的p53抑癌网络。

在脊椎动物中，miR-34家族有3个成员，即miR-34a、miR-34b和miR-34c。人miR-34a位于初始转录子的第二外显子，而miR-34b和miR-34c分别位于同一初始转录子的第一内含子和第二外显子^[19, 20]^。Xi等^[21]^通过全基因组扫描分析，发现miRNA中超过46%的启动子区有p53潜在的结合位点，说明这些miRNA有可能直接受野生型p53的调控。研究还发现miR-34家族成员与p53的状态明显相关，只有野生型p53能明显诱导miR-34的活化。在电离辐射诱导DNA损伤后，小鼠不同脏器组织中成熟的miR-34以p53依赖的方式被激活，在阿霉素诱导p53活化后，miR-34a也以依赖p53的方式明显升高，而在DOX诱导下，肺癌细胞中miR-34a的表达水平升高300多倍。miR-34b在超过90%的非小细胞肺癌中表达下降^[22]^。

### 2.4 miR-17-92与肺癌的关系

miR-17-92是由7个microRNA（miR-17-5p、miR-17-3p、miR-18a、miR-19a、miR-20、miR-19b-1和miR-92-1）组成的表达簇，是一个microRNA的多顺反子，位于染色体13q31。与正常组织相比，miR-17-92族在肺癌中表达水平明显升高，以大多数进展期的小细胞肺癌为甚^[23]^。miR-17-92族可加快肺癌细胞的生长，但其在肺癌发生发展中的作用机制尚不清楚，预测可能是通过抑制两个肿瘤抑制基因*PTEN*和*Rb2*而发挥其致癌作用。O'Donnell^[24]^研究发现miR-17-92族与小细胞肺癌中多发性高表达的致癌基因*c-Myc*相关，*Myc*基因过表达引起了miR-17-92的高表达。此外，近年来肿瘤microRNA表达谱的研究^[25]^也涉及到miR-17-92家族过表达的研究，表明在肺癌中miR-17-92家族的成员呈广泛过表达趋势。

### 2.5 Dicer酶与肺癌

Dicer是RNaseIII家族的成员，在进化上高度保守。Dicer的N端是1个RNA解旋酶结构域，随后是1个PAZ结构域，在C端是2个RNaseIII结构域和1个双链RNA结合结构域（double-stranded RNA binding domain, dsRBD）^[26]^。Dicer的作用见microRNA形成过程所述。

有研究^[27]^表明在肺腺癌的前期病变即不典型腺瘤样增生及细支气管肺泡癌中Dicer呈短暂性高表达。免疫组化和蛋白质印迹分析表明Dicer在肺鳞癌中表达高于肺腺癌，而在进展期肺腺癌中表达下调。

### 2.6 miR-128b和miR-7与肺癌

研究显示，约43%-89%非小细胞肺癌患者的肺组织标本中检测到表皮生长因子受体（epidermal growth factor receptor, EGFR）表达或高表达。大量证据^[28]^表明EGFR过度表达和/或激活与非小细胞肺癌患者生存期短、预后差、放化疗敏感性下降密切相关。胡雪君等^[29]^检测73例青年和65例老年非小细胞肺癌标本EGFR的表达，发现EGFR在非小细胞肺癌标本中的阳性表达率为51.4%。

鉴于EGFR在肺癌中的调控作用，研究人员将注意力转移到研究EGFR与microRNA的关系上来。Weiss等^[30]^挑选了miR-128b来进行此研究，因为其定位于肺癌缺失的3p22染色体上，结果显示在非小细胞肺癌细胞系中miR-128b可与EGFR的3′UTR端结合从而影响蛋白质的翻译。研究人员^[31]^发现在肺癌细胞系中miR-7也可以下调EGFR及蛋白的表达。

### 2.7 miR-107和miR-185与肺癌

细胞周期分为4期：DNA合成前期即G1期、DNA合成期即S期、DNA合成后期即G2期和细胞分裂期即M期。细胞周期调控的理论与肿瘤发生发展的关系是一个十分重要的研究领域。研究表明细胞的增殖、分化、衰老和凋亡均是细胞周期依赖性的，肿瘤细胞同样也是周期依赖性的。

2008年Kumar等^[32]^在研究多元化非小细胞肺癌小鼠模型K-RasG12D时发现，let-7g可抑制肺癌细胞的生长，使肺癌细胞周期停止或死亡。Takahashi等^[33]^将miR-107和miR-185转染至肺癌细胞中，流式细胞仪分析显示这些miRNA可使H1299细胞和A549细胞停滞在G1期，其抑制作用比let-7更强。他们定位于人类肺癌的染色体易变区域，是新的调控人类恶性肿瘤细胞的周期调节因子。

### 2.8 miR-221、miR-222与肺癌

肿瘤坏死因子相关凋亡诱导配体（TNF-related apoptosis-inducing ligand, TRAIL）是近年发现的肿瘤坏死因子（tumor necrosis factor, TNF）家族成员，与死亡受体结合后可特异性地诱导肿瘤细胞和恶性转化细胞凋亡^[34]^。Garofalo等^[35]^与Miller等^[36]^筛选出对TRAIL敏感性不同的4个非小细胞肺癌细胞系，通过比较它们在microRNA表达谱上的区别，最终证实高表达的miR-221及miR-222通过下调P27kip1抑制了TRAIL介导的细胞凋亡信号通路。*PTEN*是重要的抑癌基因之一，可以调节细胞生长和凋亡。Michela等^[37]^研究证实肺癌细胞系中miR-221及miR-222过表达，通过靶向结合PTEN 3′UTR端抑制PTEN的表达从而增加了肺癌的发生率。少量研究表明基质金属蛋白酶（matrix metalloproteinases, MMP）抑制因子TIMP3也是miR-221及miR-222下游靶标，其通过抑制TIMP3的表达而激活MMP，以增强肺癌的侵袭能力。

### 2.9 microRNA与肺癌的诊断、治疗及预后

microRNA的表达模式与人类肿瘤的诊断、分期、进展、预后等密切相关，microRNA甚至可以直接作为癌症基因治疗的靶标。

#### 2.9.1 microRNA与肺癌的诊断

许多研究表明，肺癌组织与正常肺组织的microRNA表达谱有明显的差别，越来越多的研究者将microRNA作为肿瘤的生物学标志物来探讨。Mitchell等^[38]^研究发现，在血清中可以有效分离出microRNA，并且microRNA在血清中以稳定的形式存在，无论是高温、骤冷还是强酸强碱条件下都不会被降解，这就说明血清中microRNA可以作为稳定的生物学标志物来检测癌症。Chen等^[39]^采用Solex测序方法对健康人血清和非小细胞肺癌患者血清中的小分子RNA进行测序分析。结果显示，肺癌血清与健康人血清相比microRNA表达谱明显不同，肺癌患者血清中有28种正常血清中存在的microRNA缺失，但是检测出了63种在正常血清中没有的microRNA分子。目前，对循环microRNA与肿瘤的关系研究还处于起步阶段，但是这已为肿瘤的无创诊断开辟了一条新的道路。此外，研究^[40]^还发现在福尔马林固定过的石蜡包埋组织中（甚至保存10年的标本）能够有效地检测出microRNA分子，具有广泛的临床应用前景。

#### 2.9.2 microRNA与肺癌治疗

在肺癌治疗方面，microRNA还没有得到应用，但是一些microRNA可以作为抑癌基因发挥作用，例如Trang等^[41]^通过建立小鼠非小细胞肺癌模型将let-7导入小鼠体内，结果显示let-7可以有效抑制肿瘤的生长，剔除let-7可促进小鼠肺癌的发生；尽管接受let-7治疗小鼠的肿瘤没有消失，但是66%的肿瘤均在缩小。对于抑癌基因的microRNA治疗策略是重新表达这些下调基因，理论上可以消退肿瘤。因此针对microRNA开发的microRNA相关药物不会产生如siRNA引起的完全基因表达抑制，而更接近于正常生理调节作用。

有资料认为90%的肿瘤患者死亡与肿瘤的抗药性有关，大量研究表明microRNA与肿瘤发生发展相关，也有少量研究揭示microRNA与肿瘤细胞对化疗药物的敏感程度相关。microRNA的突变、异常表达和异常加工均会影响microRNA的正常功能，导致靶基因的蛋白表达水平异常，如果此类靶基因与肿瘤细胞对药物的反应程度相关，将改变肿瘤细胞的药物敏感度。2007年Bertino等^[42]^提出了“microRNA的药物基因组学”概念，指出通过研究microRNA及microRNA的突变如何干扰microRNA的正常功能进而改变病人对药物的反应程度，最终可找出某些特定的microRNA用以指导病人的个性化用药。Shin等^[43]^发现A549细胞接受20 Gy、40 Gy放射后microRNA分别有4种、10种的改变，这些变化的microRNA与放射引起的细胞凋亡、细胞周期调控等有关，这就提示在放射治疗学中microRNA也有潜在应用价值。

#### 2.9.3 microRNA与肺癌的预后

Yanaihara等^[44]^对104例非小细胞肺癌（65例腺癌、39例鳞癌，65例Ⅰ期、17例Ⅱ期、22例Ⅲ-Ⅳ期）术后患者进行了研究，在腺癌患者中miR-155、miR-17-3p、miR-106a、miR-93高表达者和let-7a-2、miR-145、let-7b低表达者预后差。其中miR-155和let-7a-2的表达水平和患者的生存率有着密切的关系。有研究者^[45]^对112例非小细胞肺癌患者进行了检测，研究人员通过*Cox*模型和风险评分发现了5个预测非小细胞肺癌治疗效果的microRNA信号。带有这些microRNA信号的高风险基数的病人相对于低风险基数的病人，治疗效果较差。因此研究人员认为这些microRNA信号是非小细胞肺癌患者复发和存活的独立预测因子。

## 3 展望

随着基因芯片、RT-PCR和Northern blot等各种技术方法的应用和完善，检测microRNA的表达日益精确可靠，为肺癌的研究及基因诊断开辟了新的途径，也为肺癌的发生机制研究以及相关的生物治疗策略提供了新的视角。但是研究结果同时显示了microRNA作用的复杂性，如单个microRNA可以同时调控多个靶基因，而多个microRNA也可同时调控一个靶基因的表达，而且这些调控具有一定的特异性，给全面研究microRNA的功能以及构建microRNA的调控网络带来了困难。相信这些困难的解决将有利于进一步理解microRNA在生物发展中的作用，并为利用microRNA进行临床诊断和治疗提供新的依据。

## References

[1] Calin GA, Croce CM (2006). microRNA-cancer connection: the beginning of a new tale. Cancer Res.

[2] Kent OA, Mendell JT (2006). A small piece in the cancer puzzle: microRNAs as tumor suppressors and oncogenes. Oncogene.

[3] Pfeffer S, Voinnet O (2006). Viruses, microRNAs and cancer. Oncogene.

[4] Lee Y, Ahn C, Han J (2003). The nuclear RNase Ⅲ Drosha initiates microRNA processing. Nature.

[5] Lund E, Guttinger S, Calado A (2004). Nuclear export of microRNA precursors. Science.

[6] Yi R, Qin Y, Macara IG (2003). Exportin-5 mediates the nuclear export of premicroRNAs and short hairpin RNAs. Genes Dev.

[7] Khvorova A, Reynolds A, Jayasena SD (2003). Functional siRNAs and miRNAs exhibit strand bias. Cell.

[8] Lagos-Quintata M, Rauhut R, Lendeckel W (2001). Identification of novel genes coding for small expressed RNAs. Science.

[9] Ambros V, Bartel B, Bartel DP (2003). A uniform system for microRNA annotation. RNA.

[10] Calin GA, Dumitru CD, Shimizu M (2002). Frequent deletions and downregulation of microRNA gene miR15 and miR16 at 13q14 in chronic lymphocytic leukemia. Proc Natl Acad Sci USA.

[b11] 11Fang FD ed. MicroRNA methods and applications. 1st ed. Beijing: Chinese Peking Union Medical College Publication, 2008. 78-92.方福德主编. MicroRNA的研究方法与应用. 第1版. 北京: 中国协和医科大学出版社, 2008. 78-92.

[12] Cecile O, Marie PP, Marius I (2009). MicroRNAs and lung cancer: new oncogenes and tumor suppressors, new prognostic factors and potential therapeutic targets. Curr Med Chem.

[13] Takamizawa J, Konishi H, Yanagisawa K (2004). Reduced expression of the let-7 microRNAs in human lung cancers in association with shortened postoperative survival. Cancer Res.

[14] Johnson SM, Grosshans H, Shingara J (2005). RAS is regulated by the let-7 microRNA family. Cell.

[15] Fabbri M, Garzon R, Cimmino A (2007). MicroRNA-29 family reverts aberrant methylation in lung cancer by targeting DNA methyltransferases 3A and 3B. Proc Natl Acad Sci USA.

[16] Rajewsky N (2006). MicroRNA target predictions in animals. Nat Genet.

[17] Takwi A, Li Y (2009). The P53 pathway encounters the microRNA world. Current Genomics.

[18] Tarasov V, Jung P, Verdoodt B (2007). Differential regulation if microRNAs by p53 revealed by massively parallel sequencing: miR-34a is a p53 target that induces apoptosis and G1-arrest. Cell Cycle.

[19] He L, He X, Lim LP (2007). A microRNA component of the p53 tumour suppressor network. Nature.

[20] Raver-Shapira N, Marciano E, Meiri E (2007). Transcriptional activation of miR-34a contributes to p53-mediated apoptosis. Mol Cell.

[21] Xi Y, Shalgi R, Fodstad O (2006). Differentially regulated microRNAs and actively translated messenger RNA transcripts by tumor suppressor p53 in colon cancer. Clin Cancer Res.

[22] Bommer GT, Gerin I, Feng Y (2007). p53-mediated activation of miRNA34 candidate tumor-suppressor genes. Curr Biol.

[23] Hayashita Y, Osada H, Tatematsu Y (2005). A polycistronic microRNA cluster, miR-17-92, is overexpressed in human lung cancers and enhances cell proliferation. Cancer Res.

[24] O'Donnell KA, Wentzel EA, Zeller KI (2005). C-Myc-regulated microRNAs modulate E2F1 expression. Nature.

[25] Petrocca F, Visone R, Onelli MR (2008). miRiad roles for the miR-17-92 cluster in development and disease. Cell.

[26] Macrae IJ, Zhou K, Li F (2006). Structural basis for double-stranded RNA processing by dicer. Science.

[27] Chiosea S, Jelezcova E, Chandran U (2007). Overexpression of dicer in precursor lesions of lung adenocarcinoma. Cancer Res.

[28] Ciardiello F, De Vita F, Orditura M (2003). The role of EGFR inhibitors in nonsmall cell lung cancer. Eur J Cancer.

[29] Hu XJ, Song N, Liu YP (2007). Expression of EGFR, VEGF and COX-2 in non-small cell lung cancer and its significance. J Pract Oncol.

[30] Weiss GJ, Bemis LT, Nakajima E (2008). EGFR regulation by microRNA in lung cancer: correlation with clinical response and survival to gefitinib and EGFR expression in cell lines. Ann Oncol.

[31] Kefas B, Godlewski J, Comeau L (2008). microRNA-7 inhibits the epidermal growth factor receptor and the Akt pathway and is downregulated in glioblastoma. Cancer Res.

[32] Kumar MS, Erkel SJ, Pester RE (2008). Suppression of non-small cell lung tumor development by the let-7 microRNA family. Proc Natl Acad Sci USA.

[33] Takahashi Y, Forrest AR, Maeno E (2009). MiR-107 and MiR-185 can induce cell cycle arrest in human non small cell lung cancer cell lines. PLoS One.

[34] Liu Q (2009). Research advance of TRAIL molecular target in the therapy of lung cancer. Chin J Lung Cancer.

[35] Garofalo M, Quintavalle C, Di Leva G (2008). MicroRNA signatures of TRAIL resistance in human non-small cell lung cancer. Oncogene.

[36] Miller TE, Ghoshal K, Ramaswamy B (2008). MicroRNA-221/222 confer trmoxifen resistance in breast cancer by targeting P27 (Kip1). J Biol Chem.

[37] Michela G, Gianpiero DL, Giulia R (2009). miR-221 & 222 regulate TRAIL resistance and enhance tumorigenicity through PTEN and TIMP3 downregulation. Cancer cell.

[38] Mitchell PS, Parkin RK, Kroh EM (2008). Circulating microRNAs as stable blood-based markers for cancer detection. Proc Natl Acad Sci USA.

[39] Chen X, Ba Y, Ma L (2008). Characterization of microRNAs in serum: a novel class of biomarkers for diagnosis of cancer and other diseases. Cell Res.

[40] Siebolts U, Varnholt H, Drebber U (2009). Tissues from routine pathology archives are suitable for microRNA analyses by quantitative PCR. J Clin Pathol.

[41] Trang P, Medina PP, Wiggins JF (2009). Regression of murine lung tumors by the let-7 microRNA. Oncogene.

[42] Bertino JR, Banerjee D, Mishra PJ (2007). Pharmacogenomics of microRNA: a miRSNP towards individualized therapy. Pharmacogenomics.

[43] Shin S, Cha HJ, Lee EM (2009). Alteration of miRNA profiles by ionizing radiation in A549 human non-small cell lung cancer cells. Int J Oncol.

[44] Yanaihara N, Caplen N, Bowman E (2006). Unique microRNA molecular profiles in lung cancer diagnosis and prognosis. Cancer Cell.

[45] Yu SL, Chen HY, Chang GC (2008). MicroRNA signature predicts survival and relapse in lung cancer. Cancer Cell.

